# Complete Genome Sequence of Mycobacteriophage IgnatiusPatJac

**DOI:** 10.1128/mra.00664-22

**Published:** 2022-09-21

**Authors:** Olivia Jacobs, Nikki Gentle, Christopher Ealand, Bavesh Kana

**Affiliations:** a DSI/NRF Centre of Excellence for Biomedical TB Research, Faculty of Health Sciences, University of the Witwatersrand, National Health Laboratory Service, Johannesburg, South Africa; b School of Molecular and Cell Biology, Faculty of Science, University of the Witwatersrand, Johannesburg, South Africa; Loyola University Chicago

## Abstract

IgnatiusPatJac is a *Siphoviridae* mycobacteriophage capable of lytic infection in Mycobacterium smegmatis and Mycobacterium tuberculosis. It was isolated from damp soil in Johannesburg, South Africa. The 51,164-bp double-stranded DNA genome has a GC content of 63.6%, predicted to encode 93 genes. IgnatiusPatJac is classified as an A1 subcluster mycobacteriophage.

## ANNOUNCEMENT

Mycobacterium tuberculosis (*Mtb*), the causative agent of tuberculosis (TB), remains one of the most successful pathogens (1). In Southern Africa, HIV and TB coinfection exacerbates the burden of disease (2). The emergence of drug-resistant TB has necessitated the development of novel therapeutics, including alternative TB treatment strategies such as lytic mycobacteriophages ([Bibr B3][Bibr B4][Bibr B6]). We isolated IgnatiusPatJac from a damp soil sample collected at a construction site in Johannesburg, South Africa (16 April 2021; GPS coordinates −26.01014 and 27.98844).

**FIG 1:**
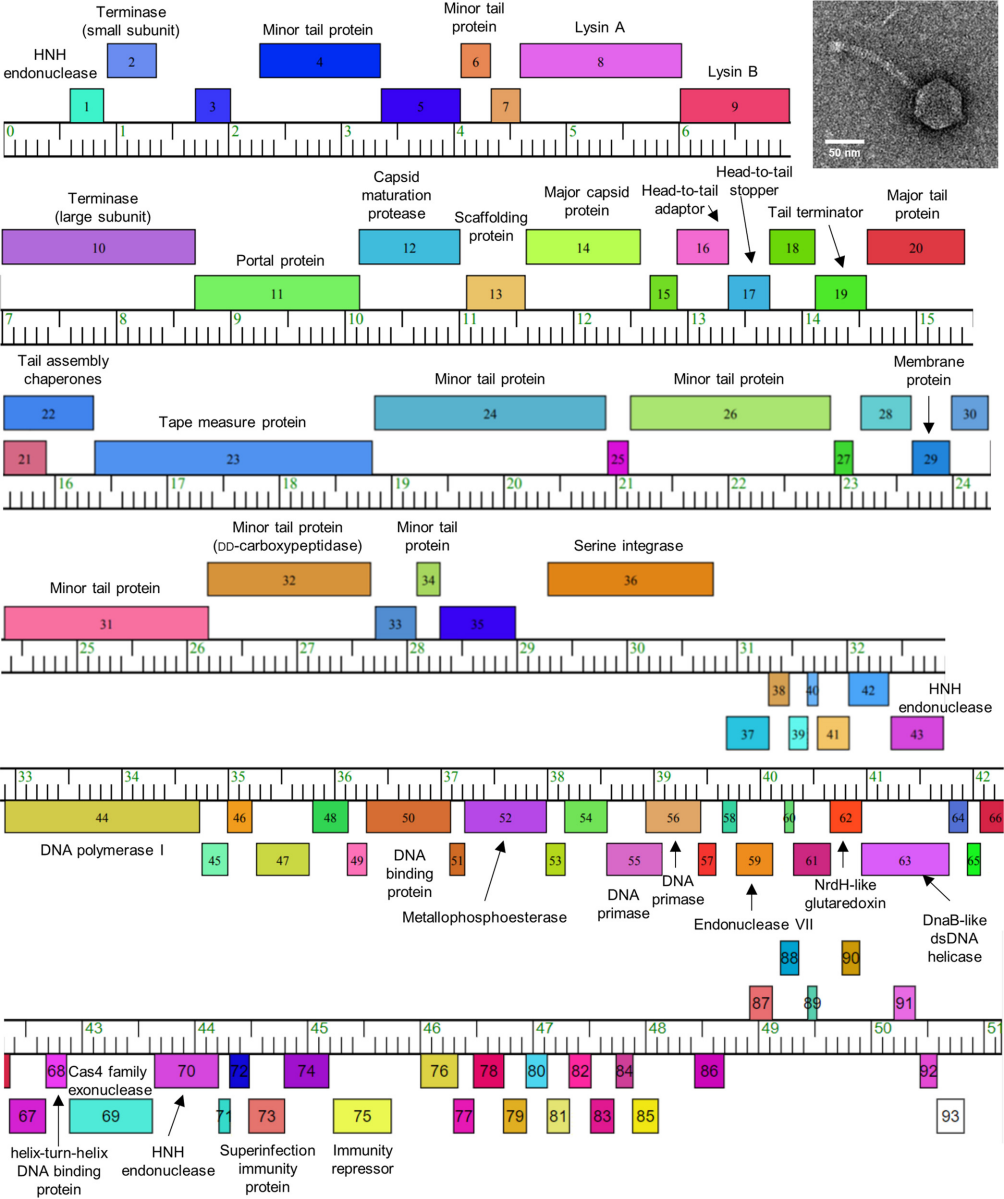
Genome organization of mycobacteriophage IgnatiusPatJac. The double-stranded DNA genome of IgnatiusPatJac is represented as a horizontal bar with vertical markers at every kilobase pair. Gene numbers are shown in boxes, which are shaded according to their phamily assignments determined using Phamerator (7). Boxes above and below represent transcription from plus and negative strands, respectively. Putative gene functions, based on BLAST analyses, are indicated. The inset represents a transmission electron micrograph of IgnatiusPatJac (scale bar equivalent to 50 nm). Droplets of purified IgnatiusPatJac lysate were placed onto copper grids (Agar Scientific, UK), coated with carbon, and rendered hydrophilic using an EMS100 Glow Discharge Unit (Electron Microscopy Sciences, USA). Samples were visualized using negative staining with 2% uranyl acetate (SPI Supplies, USA).

Soil samples were washed with phage (MP) buffer and mycobacteriophages purified through a 0.22-μm filter. For infection, 50 μL of filtrate was incubated with stationary-phase Mycobacterium smegmatis mc^2^155 for 48 h at 37°C (7, 8). Emergent plaques were picked for mycobacteriophage purification. Negative staining transmission electron microscopy revealed that IgnatiusPatJac has a *Siphoviridae* morphology, with an icosahedral head diameter of ~60 nm and a tail length of ~120 nm (Fig. 1).

Genomic DNA was isolated using the Wizard Genomic DNA purification kit (Promega). DNA was then sheared to ~10-kb fragments using g-tubes (Covaris). SMRTbell libraries were constructed using PacBio’s Microbial Multiplexing workflow and size-selected for fragments between 10 and 18 kb using BluePippin with U1 marker (Sage Science). Libraries were indexed with the Barcoded Overhang Adapter kit 8A/8B (PacBio), prepared using the online SMRTlink guided protocol (Binding kit 2.0 and Control 1.0; Sequel II sequencing plate 2.0 and SMRT cell 8M) and sequenced on the Sequel IIe (PacBio) HiFi platform. A total of 490 single-end reads were obtained with an average read length (*N*_50_) of 6116 bp. Raw reads were assembled using the “Genome Assembly” application with SMRT link (v10.1.0.119588) using default settings. A single mycobacteriophage contig was assembled and checked for quality, completeness, accuracy, and mycobacteriophage genomic termini using Consed (v29.0) (9).

IgnatiusPatJac contains a circularly permuted genome of 51,164 bp, a 3’ sticky overhang of 10 bp (CGGACGGTAA), and a GC content of 63.6%. The approximate coverage level by CCS (circular consensus sequencing) was 45-fold. Whole-genome nucleotide BLAST alignments (https://blast.ncbi.nlm.nih.gov/) showed 96.72% nucleotide similarity to both the cluster A1 mycobacteriophages Moose (GenBank accession number MH479919.1) and Forsyhteast (MG925342.1). Auto-annotation was performed using GeneMark (v2.5p) (10) and Glimmer (v3.07) (11) followed by manual revision with DNA Master (v5.23.6; http://phagesdb.org/DNAMaster/), Phamerator (https://phamerator.org) (12), and HHPRED (https://toolkit.tuebingen.mpg.de/tools/hhpred) (13). Default parameters were used for all software tools.

The IgnatiusPatjac genome is predicted to encode 93 open reading frames (ORFs) with no tRNA or transfer-mRNA (tmRNA) genes detected using ARAGORN and tRNAscan-SE (14, 15) (Fig. 1). Of the 93 predicted ORFs, 54 (58.1%) were annotated as hypothetical proteins. ORFs 1 to 36 and 87 to 93 are transcribed from the positive strand. These regions include structural and assembly proteins, serine integrase, and a lysis cassette encoding Lysin A and B genes. The tail assembly chaperones (ORFs 21 and 22) have a −1 frameshift. In contrast, ORFs 37 to 86, transcribed from the negative strand, encode several proteins, including HNH endonuclease, DNA polymerase I, metallophosphoesterase, two DNA primases, endonuclease IV, NrdH-like glutaredoxin, immunity repressor proteins, and DnaB-like dsDNA helicase.

### Data availability.

The IgnatiusPatJac genome sequence is available at GenBank under accession number ON677304. The raw sequencing reads are available in the SRA under accession number SRX15605400.
